# Optimization of Glycerol–Water Extraction of Selected Bioactive Compounds from Peppermint and Common Nettle

**DOI:** 10.3390/antiox10050817

**Published:** 2021-05-20

**Authors:** Grażyna Kowalska, Tomasz Baj, Radosław Kowalski, Jolanta Szymańska

**Affiliations:** 1 Department of Tourism and Recreation, University of Life Sciences in Lublin, 15 Akademicka Str., 20-950 Lublin, Poland; grazyna.kowalska@up.lublin.pl; 2 Department of Pharmacognosy, Medical University of Lublin, 1 Chodźki Str., 20-093 Lublin, Poland; tomasz.baj@umlub.pl; 3 Department of Analysis and Food Quality Assessment, University of Life Sciences in Lublin, 8 Skromna Str., 20-704 Lublin, Poland; 4 Department of Integrated Paediatric Dentistry, Chair of Integrated Dentistry, Medical University of Lublin, 6 Chodźki Str., 20-093 Lublin, Poland; jolanta.szymanska@umlub.pl

**Keywords:** glycerol, extraction, polyphenols, flavonoids, chlorophyll

## Abstract

Current trends in the industry indicate that extraction solvents should conform with the ideals of so-called “green chemistry”. Therefore, the objective of the presented study was to optimize the conditions for the extraction of polyphenols, flavonoids and chlorophyll from peppermint leaves (*Mentha* × *piperita* L., Lamiaceae) and from common nettle leaves (*Urtica dioica* L., Urticaceae) via green chemistry. The obtained experimental results were subjected to modelling by means of the multiple regression method, while the optimization of the system was addressed via the application of the desirability function. As a result of the use of glycerol–water systems for the extraction of the tested active compounds from mint leaves and nettle leaves, extracts with higher concentrations of polyphenols, flavonoids and chlorophyll were most often obtained, when compared with the use of classical solvents such as water and ethanol. In this work, we demonstrate that the extraction temperature has significant influence on the concentration of the determined components in the extracts. To obtain the highest values of the analysed parameters, leaves of peppermint should be extracted with glycerol–water mixture at the proportions of 30.5:69.5 at a temperature of 50 °C, while the optimal conditions for the extraction of leaves of common nettle were the glycerol–water proportions of 12.5:87.5 and extraction temperature of 20 °C. Comparing the average percentage differences between the highest values of the analysed parameters obtained in the experiment and the approximated values for various temperatures with the level of desirability, one can note a high correlation that, in the analysed examples, amounted to 0.9681. The study showed that glycerol can be an alternative solvent in the extraction of polyphenols, flavonoids and chlorophyll, replacing, e.g., ethanol—which, for various reasons, cannot always be used.

## 1. Introduction

The application of herbs in pharmacology or cooking is determined by the number and variety of biologically active substances contained within them. Today’s pharmacologists, however, are searching for convenient alternatives to unprocessed herbs, and in this case, these are extracts from plants that can either be used directly or as semi-products which, when subjected to further unit processing, will become components of ready products characterised by valuable utility values. The classic liquid extracts, and especially extracts with consumable characteristics, are obtained with the use of known edible polar (i.e., water and ethanol) and apolar (i.e., vegetable oils) solvents [1]. The choice of solvent is related to its extraction capacity relative to the chemical character of a specific group of compounds but should also take into account its preserving properties. This is because these values have an impact on the shelf life of the products. Beyond the availability of the solvent and its market price, other important aspects include the health status and age of the consumer of the extracts. Among other issues, this includes ethanol content in products for non-adults, for persons addicted to alcohol or drivers and operators of various vehicles and mobile machinery, or sugar in products that can be consumed by diabetics.

Polyphenols are an important group of biologically active plant compounds with a wide range of applications. The antioxidant activity of polyphenols is related to their effect on the formation and proliferation of free radicals and results from the presence of –OH groups. Here, the antioxidant properties increase with increase of their number in the molecule [2].

Polar solvents, to which –OH groups will display a greater affinity, allow for obtaining extracts with a higher antioxidant potential than apolar solvents. Indeed, Arumugam et al., [2] demonstrated that the antioxidant properties of solutions obtained from mint through water extraction were 3.5-fold higher than those obtained with the use of acetone and hexane [2].

The positive effect of temperature on the efficiency of extraction of phenolic compounds has also been researched extensively [3,4,5,6,7,8]. The cited authors report that the increase of extraction efficiency is related both to increase of solubility of phenolic compounds in the solvent and with intensification of the processes of diffusion of phenolic compounds from inside plant cells to the surrounding medium, i.e., the solvent. Vuong et al., [6] noted that extraction proceeded most efficiently in the temperature range of 50–70 °C, and when temperatures of 90–100 °C were attained, there was a sudden drop of extraction efficiency. This was believed to be related with the thermal degradation of polyphenolic molecules.

Industrial and laboratory extraction is conducted, most frequently, with the use of solvents, such as water, ethyl alcohol, acetone, hexane and chloroform, and, therefore, most studies concerned with the isolation of bioactive substances from plant material also involve the use of the aforementioned solvents. However, literature data are scarce concerning the use of glycerol for the same purpose. This polyalcohol has a sweet taste and a low glycaemic index [9]. Glycerol is used in pharmacy and in food technology; it is characterised by interesting utility features, and, at concentrations applied in these branches of industry, it is not toxic to humans [9]. As opposed to ethanol, glycerol is not flammable and displays low volatility, but at the same time, like ethanol, it easily mixes with water, which is one of the properties that can be used for the extraction of active substances with health promoting properties.

The employment of glycerol for the preparation of plant extracts, however, is not as common as the use of water or ethanol. Taking into account the problem of utilization of by-products formed during various key production technologies (e.g., glycerol in the production of biodiesel), it is recommended to search for new applications for them or to intensify those already known, which falls within the scope of the so-called “green chemistry” [9].

Glycerol–water systems are being exploited for the acquisition of valuable components from plant waste originating from various industrial technologies [10]. Indeed, the spectrum of application of that solvent has expanded into the extraction of polyphenols [11,12,13,14]. As stated in Eyiz et al., [11], its mixtures with water allow for obtaining plant extracts, which have value due to their content of phytochemical compounds such as phenols, flavonoids, anthocyanins, proanthocyanidins and ascorbic acid.

The harnessing of the properties of glycerol is particularly up to date in relation to “green chemistry”, to which the use of the solvent for the acquisition of natural phytocompounds can be referenced [15,16,17,18]. Glycerol is characterised by low price, ease of acquisition and fitness for use for consumption purposes.

Many plants are known to provide important ingredients for industrial production. The most interesting industrial plants are widely introduced into cultivation, constituting a significant share in herbal production. Two key species grown for industrial purposes are peppermint and nettle. These two species provide raw materials that are widely used in the pharmaceutical, food and cosmetic industries. This has come about because of the well-established tradition of using these plants in folk medicine, and this has been confirmed by multidirectional research [19,20,21,22]. Both peppermint and nettle extracts contain valuable active compounds such as polyphenols (phenolic acids—especially caffeic acid and their esters—depsides, rosmarinic acid), flavonoids (eriocitrin, luteolin-7-*O*-rutinoside, hesperidin, diosmin, rutin), volatile compounds (menthol, menthone and other possible constituents such as pulegone and menthofuran), chlorophyll, carotenoids and vitamins and minerals (K, Ca, Mg, Fe, Cu, Mn, Zn, B) [23,24], and there are numerous products on the market containing peppermint and nettle extracts [23,25]. The industrial demand for these raw materials has stimulated the interest of agricultural producers, who have responded by increasing the acreage of cultivation of these species.

Peppermint, *Mentha piperita* L., from the family Lamiaceae, is commonly grown in Europe and in North America and it is one of the main herbal plants grown in Poland. The main raw material is the leaf of peppermint—*Folium Menthae piperitae* [26]. The leaf contains up to 2% of essential oil, with menthol as the primary component. Other components include polyphenols, i.e., tannins, phenolic acids, flavonoids and bitters [26]. Extracts from peppermint leaf stimulate the secretion of gastric juice and have choleretic- and bile-forming properties, through which they facilitate digestion and assimilation of food. In addition, products containing extracts from peppermint have a diastolic effect and reduce the tension of smooth muscles of the intestines and bile and urinary tracts, intensify the peristaltic movements of the intestines and prevent meteorism [19,26]. Components present in peppermint are also characterised by antibacterial activity [27,28].

Common nettle, *Urtica dioica* L., from the family Urticaceae, can be found growing wild almost all over the world and especially in the moderate climate zone. It is treated as a common weed, but it is also known as a utility plant and has medicinal, edible, fodder and cosmetic applications. In medical usage, we employ the herbage—*Herba Utricae dioicae*, leaves—*Folium Urticae* and roots—*Radix Urticae* of common nettle [29]. Nettle leaf is an important source of chlorophyll, which can be exploited, e.g., as a pigment in food and pharmaceutical products or in cosmetics. In addition, its raw material contains polyphenolic compounds, carotenoids, organic acids, vitamins B2, K, C, E and mineral components [29]. Extracts from nettle leaf have diuretic, detoxifying, anti-inflammatory, antidiabetic, disinfecting and bacteriostatic effects. They increase the level of haemoglobin in the plasma, as well as the number of red blood cells, stimulate the secretion of gastric juice, have a choleretic effect and also decrease and regulate blood pressure. Moreover, they have a mollifying effect in inflammations of the skin, stimulate hair bulbs and have anti-dandruff and anti-seborrheic properties [29,30].

The possibility of using post-extraction or after hydrodistillation plant material plays an important role in the valorisation of active compounds. Peppermint leaves, after distillation of essential oil, still contain the polyphenols, which may be an additional perspective for consideration in the frame of the valorisation of agro-food residues [31]. In addition, the prospect of sourcing peppermint essential oil is important. The post-extraction material can be used to recover the essential oil. Previous studies have shown that 34.85% to 66.67% of the original amount of mint essential oil can be recovered from the post-extraction plant material (after the brewing process) [32].

Particular attention should be paid to the polyphenols and chlorophyll pigments contained in nettle and peppermint products. The protective role of polyphenols against reactive oxygen and nitrogen species, UV rays, plant pathogens, parasites and predators can be used in prophylaxis or can help recovery from serious human diseases, especially from various types of cancer [33]. Ubiquity, specificity of the response and the absence or low toxicity are key advantages of the use of polyphenols as anti-cancer agents [33]. The presence of natural pigments in our daily diet, such as chlorophyll or carotenoids, results from the increased awareness of the need to introduce fresh vegetables and fruits into common human consumption [34]. There are reports of the many positive biological functions of enhanced chlorophyll intake in the diet. The best known is probably the ability to capture mutagens due to the flat structure of chlorophylls, which reduces the availability of harmful compounds in the cell [34].

Plant-derived substances are extensively used worldwide. They find applications in the pharmaceutical, food, cosmetic and other industries. For this reason, one of the fundamental challenges the industry is facing is to develop optimum methods of extraction such that the use of the simplest processes possible will allow for achieving the maximum yield of active substances. For this purpose, various mathematical modelling approaches are employed. The optimisation can relate both to the conditions of the process of extraction and to the suitable choice of solvents. The mixture design model is more and more frequently applied for the development of, e.g., the composition of mixtures with optimum microbiological and biological properties [35,36] and the composition of extraction solvents [37].

The objective of the presented study is the optimization of the conditions for the extraction of polyphenols, flavonoids and chlorophyll from peppermint and nettle leaves by employing the properties of glycerol–water systems. This is a pioneering study. The need to conduct such research results from the lack of data available in the literature on the optimization of extraction with the use of glycerol for the abovementioned plant materials. This will enable wider use of glycerol systems for obtaining various extracts of peppermint and nettle leaves that could be alternatives for the commonly used water or ethanol extracts.

## 2. Materials and Methods

### 2.1. Experimental Material

The following air-dried plant materials were used for the analyses: leaf of peppermint *Mentha* × *piperita* L. (FLOS, Mokrsko, Poland) and leaf of common nettle *Urtica dioica* L. (FLOS, Mokrsko, Poland). These were derived from the production batch of 2018. The plant material was standardised through pooling together and fragmentation to a homogeneous fraction, after which the moisture level of each product was measured for conversion to dry matter using a moisture analyser type WPS 50 SX (RADWAG, Poland, Radom).

In addition, the extracts were prepared with the use of plant glycerol with pharmaceutical purity grade (99.5%, TechlandLab, Tarnobrzeg, Poland).

### 2.2. Water Extraction and Glycerol–Water Extraction

Weighed portions (2.0 g) of leaf of peppermint and common nettle were placed in a conical flask with volume of 250 mL. Next, a suitable extraction solvent (100 mL, with temperature of 20 °C, 50 °C or 80 °C), prepared in accordance with Table 1, was added to the plant material. The process of extraction was conducted in a temperature-controller shaker for 10 min, maintaining the temperature at the pre-set levels. Samples of 50 mL were collected from the extracts and then strained through filter paper, and the filtrate was used for further assays. Every extract was prepared in triplicate.

### 2.3. Ethanol Extraction and Ethanol–Water Extraction

The reference extracts for the glycerol–water extracts were conventional extracts obtained with the use of ethanol (98.8%—E) and with the use of an ethanol–water system (50%—E 50%).

### 2.4. Evaluation of Total Polyphenols, Flavonoids and Chlorophyll Contents

Assessments of total phenolic compounds (with conversion for gallic acid GA) in the tested macerates were made by spectrophotometric means (λ = 725 nm) according to a modified method [38]. The results were calculated from the equation of the calibration curve prepared for gallic acid standards (Sigma–Aldrich, St. Louis, MO, USA; ACS reagent ≥98.00%) in the concentration range of 10–60 mg/L (10, 20, 30, 40, 50, 60 mg/L). Each sample, depending on the herbal material, was diluted appropriately to the range of the standard curve.

Evaluation of total flavonoid content (flavonoles converted for quercetin Q equivalents) in the tested macerates was performed by use of spectrophotometry (λ = 425 nm) according to a modified procedure [39]. The results were calculated from the equation of the calibration curve prepared for quercetin standards (Sigma–Aldrich, St. Louis, MO, USA; ACS reagent ≥98.00%) in the concentration range 12–120 mg/L (12, 18, 30, 90, 120 mg/L). Each sample, depending on the herbal material, was diluted appropriately to the range of the standard curve.

Evaluation of total chlorophyll content (sum of chlorophylls a and b) was conducted by means of the spectrophotometric method at wavelength of λ = 664 nm (chlorophyll a) and λ = 648 nm (chlorophyll b) according to the modified procedure of Lichtenthaler and Buschmann [40].

All analyses were performed in triplicate. Additionally, the concentrations of polyphenols, flavonoids and total chlorophyll in the obtained extracts were converted into the weight of dry plant material [mg/g DM].

### 2.5. Evaluation of Total Antioxidant Capacity Based on Ferric Ion (III) Reduction (FRAP)

The ferric ion reducing antioxidant parameter (FRAP) method allows us to determine the properties or ferric ion reduction by the analysed substance, which is the measure of its antioxidant properties. At a low pH (ensuring the solubility of Fe^+3^ salts), under the effect of substances with antioxidant properties, ferric ions in a complex with TPTZ (2,4,6-tripyridil-s-1,3,5-triazine) (Fe^+3^—TPTZ) undergo reduction to Fe^+2^. The reaction proceeds with simultaneous development of blue colouring, the change of which is analysed by measuring the absorbance of the solution at wavelength of λ = 593 nm. In this study, a modification of a method described earlier was employed [41].

### 2.6. Optimal Parameters of the Extraction Mixture

In the presented experiment, the experimental results obtained were subjected to modelling with the use of the multiple regression method, and the optimisation of the system was addressed with the application of the desirability function.

For the estimation of the optimum values of the two-component system, glycerol:water, a quadratic polynomial model (second-order) for two independent variables was employed, as described by the formula (1) below [42]:

Y = *β*_0_ + *β* _1_*x*_1_ + *β* _2_*x*_2_ + *β*_11_*x*_1_^2^ + *β* _22_*x*_2_^2^ + *β* _12_*x*_1_*x*_2_ (1)

where Y is the dependent variable, *x*_1_, *x*_2_ are independent variables, and *β*_1_… *β*_12_ are the regression coefficients.

In the solution proposed by Derringer and Suich in 1980, it was assumed that quality is based on the value of “desirability”, which falls within specific limits [43]. The method, deemed the “desirability function”, includes the transformation of the expected response Y into a dimensionless function of fragmentary desirability *d_i_*. The permissible upper limit (*U_i_*) is 1, and the lower undesirable limit (*L_i_*) assumes the value of 0 [43]. The maximum response of the above can be written as below (2):):

(2) $$d_{i}\;\left({\hat{y}}_{i}\left(x\right)\right)\;=\;\left(\begin{array}{cc} 0 & \mathrm{if}\;{\hat{y}}_{i}\left(x\right)\;<\;L_{i}\\ \left(\frac{{\hat{y}}_{i}\left(x\right)\;-\;L_{i}}{U_{i}\;-\;L_{i}}\right) & \mathrm{if}\;L_{i}\;\leq \;{\hat{y}}_{i}\left(x\right)\;\leq U_{i}\\ 1 & \mathrm{if}\;{\hat{y}}_{i}\left(x\right)\;>\;U_{i} \end{array}\right)$$

where *s* is the value of power with the name “weight” and *L_i_* and *U_i_* are the lowest and the highest level of limit for response *i*, respectively. The optimisation of extraction processes can be performed with the use of the desirability function.

For the purpose of optimizing many parameters of extraction, the function called the “global desirability” (*D*) is employed. This is defined as the geometric mean of various values di [44] (3):

*D* = [*d*_1_^*p*_1_^ × *d*_2_^*p*_2_^ × *d*_3_^*p*_3_^ × … × *d*_*n*_^*pn*^] ^1/^^Σ^^ni^ (3)

where *p_i_* is the weight of every variable relative to the other variables.

The optimisation was performed simultaneously for the 4 parameters of the sums of polyphenols, flavonoids, chlorophyll, and the antioxidant activity, using the experimental results for the concentration of those components obtained for the glycerol systems.

### 2.7. Statistical Analysis

Data were analysed by way of applying one-way ANOVA, followed by Duncan’s test (*p* ≤ 0.05), through the use of the SAS statistical system (SAS Version 9.1, SAS Inst., Cary, NC, USA.). The optimization was carried out by employing the Statistica 12.0 package (StatSoft, Tulsa, OK, USA).

## 3. Results and Discussion

### 3.1. Total Polyphenols, Flavonoids and Chlorophyll Contents of Extracts from Peppermint and Common Nettle

Table 2 and Table 3 present the results of determination of the content of total polyphenols, flavonoids and chlorophyll in the water, glycerol and ethanol extracts obtained in the presented experiment.

#### 3.1.1. Polyphenols

Extracts from peppermint leaves contained higher levels of polyphenols as converted to gallic acid GAE (up to 412.5 mg GAE/L) when compared with extracts from nettle leaves (up to 161.8 mg GAE/L). The content of polyphenols in water extracts from peppermint and nettle was 387.7 mg GAE/L and 113.9 mg GAE/L (80 °C); 357.1 mg GAE/L and 120.3 mg GAE/L (50 °C); and 310.9 mg GAE/L and 91.4 mg GAE/L (20 °C), respectively. In contrast, the concentration of polyphenols in anhydrous ethanol extracts from peppermint and nettle was on the level of 40.7 mg GAE/L and 31.9 mg GAE/L (50 °C) and 29.5 mg GAE/L and 22.3 mg GAE/L (20 °C), respectively. Ethanol–water systems (50%) were better extraction solvents when compared with anhydrous alcohol, allowing for significant increase in the concentration of polyphenols in peppermint and nettle extracts—for extraction conducted at temperature of 50 °C: to 319.6 mg GAE/L (ca. 9-fold increase relative to anhydrous ethanol) and to 109.0 mg GAE/L (ca. 3-fold increase relative to anhydrous ethanol); and for extraction at 20 °C: to 286.2 mg GAE/L (ca. 10-fold increase relative to anhydrous ethanol) and to 77.9 mg GAE/L (ca. 3-fold increase relative to anhydrous ethanol).

The application of glycerol–water systems for the extraction of the polyphenolic fraction from leaves of peppermint and nettle allowed for obtaining various concentrations of polyphenols depending on the percentage concentration of glycerol and on the temperature of extraction.

In the case of peppermint leaf extraction conducted at temperature of 80 °C, the most efficient extraction solvent in terms of the content of polyphenols was a 30% solution of glycerol. This allowed us to obtain an extract with the concentration of 412.5 mg GAE/L, which, statistically, was equivalent to the water extract. An increase in the concentration of glycerol to 50%, 65% and 80%, respectively, however, brought about a decrease in the concentration of polyphenols to 372.4 mg GAE/L, 340.7 mg GAE/L and 276.7 mg GAE/L. Peppermint leaf extraction with glycerol–water systems at temperature of 50 °C and 20 °C was also characterised by a decrease of the level of polyphenols concentration in the extracts.

In the case of nettle leaf extraction with glycerol–water systems, we observed that a decrease of extraction temperature systems with a higher concentration of glycerol were characterised by a lower efficiency. At a temperature of 80 °C, systems with glycerol concentration from 30% to 65% had similar extraction properties relative to polyphenols isolated from nettle leaves. Extraction conducted at temperature of 50 °C was the most efficient for the glycerol–water systems of 30% and 50%. In the case of extraction conducted at temperature of 20 °C, only the glycerol–water system was optimal and allowed for obtaining the highest concentration of polyphenols from nettle leaves (100.3 mg GAE/L) as compared with all other extraction solvent variants applied (from 22.3 mg GAE/L—anhydrous ethanol, to 91.4 mg GAE/L—water).

**Figure 1:**
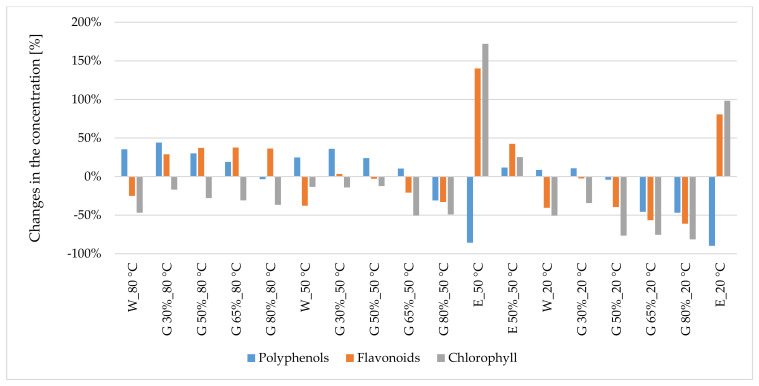
Changes (%) in the concentration of polyphenols, flavonoids and chlorophyll in extracts from peppermint leaves compared with 50% ethanol–water extract (20 °C). W—water, G—glycerol. Sample code—designations as in Table 1.

**Figure 2:**
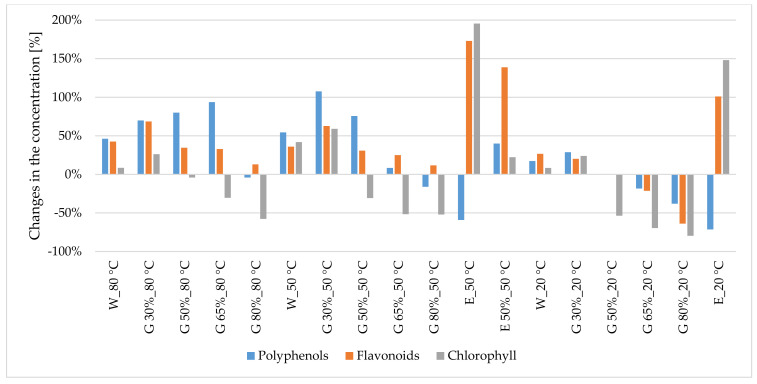
Changes (%) in the concentration of polyphenols, flavonoids and chlorophyll in extracts from common nettle leaves compared with 50% ethanol–water extract (20 °C). W—water, G—glycerol. Sample code—designations as in Table 1.

Overall, comparing percentage changes in the content of polyphenols in glycerol–water extracts from peppermint and nettle to the corresponding 50% ethanol–water extracts (20 °C), an increase is observed in the concentration of those components in extracts prepared at temperatures of 80 °C and 50 °C (Figure 1 and Figure 2).

The analysis of the data available in the literature on the content of polyphenols in peppermint leaves and nettle leaves shows a large diversity of the obtained results. This depends, among other reasons, on botanical features and cultivation factors, processing technology and conditions of the extraction process [45,46,47]. In the present study, the following results were obtained for the content of active substances per gram of dry plant material: in mint leaves—from 1.5 to 20.6 mg GAE/g DM, in nettle leaves—from 1.1 to 8.1 mg GAE/g DM. However, according to the literature, the content of polyphenols in peppermint and nettle is, respectively, in the range of: 11.56–27.12 mg/g DM (two-step extraction with 50% methanol) [45], from 5.58 to 63 mg/g DM (80% methanol extraction with sonication) [46], from 27.08 to 35.28 mg/g DM (water extraction and methanol extraction) [48], from 4.21 to 6.43 mg/g DM (70% methanol extraction) [49] and 7.62 mg/g DM (70% ethanol extraction) [50], 2.49 mg/g DM and 7.89 mg/g DM (chloroform, 80% methanol and water extractions) [51], from 6.13 to 11.62 mg/g DM (SFE with carbon dioxide and 80% ethanol extraction) [52], from 2.9 to 5.1 mg/g DM (96% ethanol extraction) [53], 10.95 mg/g DM (water and water–ethanol extraction) [54]. The results obtained in our study are within the range of data available in the literature.

#### 3.1.2. Flavonoids

Higher concentrations of flavonoids, as converted to quercetin QE, were noted in extracts from leaves of peppermint (up to 199.8 mg QE/L) as compared with extracts from leaves of common nettle (up to 102.7 mg QE/L). The content of flavonoids in water extracts from peppermint and nettle was 62.3 mg QE/L and 53.6 mg QE/L (80 °C), 51.7 mg QE/L and 51.1 mg QE/L (50 °C), 49.5 mg QE/L and 47.6 mg QE/L (20 °C), respectively. In contrast, the concentration of flavonoids in anhydrous ethanol extracts from peppermint and nettle was at the levels of 199.8 mg QE/L and 102.7 mg QE/L (50 °C), 150.3 mg QE/L and 75.6 mg QE/L (20 °C), respectively. This was the optimal extraction system among the extraction solvents used in the experiment. The ethanol–water systems (50%) were found to be less efficient extraction solvents compared to anhydrous ethanol, yielding flavonoid concentrations in peppermint and nettle extracts as follows: for extraction conducted at temperature of 50 °C: 18.5 mg QE/L and 89.8 mg QE/L and for extraction conducted at temperature of 20 °C: 83.2 mg QE/L and 37.6 mg QE/L.

On applying glycerol–water systems for the extraction of the flavonoid fraction from leaves of peppermint and nettle, most frequently, extracts with higher concentration of flavonoids were obtained, compared with water extracts. In peppermint leaves extraction conducted at temperature of 80 °C, all glycerol–water systems displayed similar extraction properties, producing extracts with flavonoid concentration in the range from 107.2 mg QE/L to 114.5 mg QE/L. We also saw that glycerol concentrations of 30%, 50% and 65% yielded significantly the highest efficiency in terms of flavonoid content in common nettle extracts for the temperature of 80 °C.

At the temperature of 20 °C, the most efficient extraction solvent in relation to flavonoids was the 30% glycerol system. This allowed us to obtain flavonoid concentrations in peppermint and nettle extracts at 81.1 mg QE/L and 45.2 mg QE/L, respectively. An increase of the percentage content of glycerol relative to water resulted in a lower flavonoid extraction efficiency from the analysed plant materials.

Comparing the percentage changes in the content of flavonoids in glycerol–water extracts from peppermint and nettle with the corresponding 50% ethanol–water extracts (20 °C), one can observe an increase in the concentration of those components in the glycerol extracts (Figure 1 and Figure 2).

In this study, the following results were obtained for the total flavonoid content per gram of dry plant material: in mint leaves—from 1.6 to 10.0 mg QE/g DM, and in nettle leaves—from 0.7 to 5.1 mg QE/g DM. The literature states that the content of flavonoids in peppermint ranges from 12.5 to 25.95 mg/g DM (80% methanol extraction with sonication) [46], from 3.8 to 6.7 mg/g DM (70% methanol extraction) [55], from 0.62 to 2.13 mg/g DM (70% methanol extraction) [49]. In the case of the content of flavonoids in nettle leaves, the data in the literature are as follows: 20.29 mg/g DM (80% ethanol extraction) [56], 3.68–4.10 mg/g DM (SFE with carbon dioxide and 80% ethanol extraction) [52], 4.84 and 15.40 mg/g DM (chloroform, 80% methanol and water extractions) [51], 6.90 mg/g DM (water and water–ethanol extraction) [54]. On comparing the experimental results with the literature data on the content of flavonoids in the tested plant materials, it can be concluded that they confirm the previous research results.

#### 3.1.3. Chlorophyll

The highest concentration of chlorophyll was noted in anhydrous ethanol extracts, the respective levels for peppermint and nettle being 276.2 mg/L and 354.7 mg/L (50 °C), and 201.3 mg/L and 297.7 mg/L (20 °C). Moreover, the ethanol–water extracts were characterised by significantly high concentrations of chlorophyll, at 127.1 mg/L and 190.9 mg/L (50 °C) and 101.5 mg/L and 148.7 mg/L (20 °C), respectively, for peppermint and nettle. The glycerol–water extraction systems were characterised by diversified extraction properties. The highest concentrations of chlorophyll were noted in glycerol extracts in which the content of glycerol was 30%, where the concentrations for peppermint and nettle, respectively, were 84.4 mg/L and 151.4 mg/L (80 °C); 88.0 mg/L and 170.2 mg/L (50 °C); and 66.6 mg/L and 130.1 mg/L (20 °C). An increase in the content of glycerol in the extraction systems involved a reduction of the extraction properties relative to chlorophyll.

The total chlorophyll content per gram of dry plant material was as follows: in mint leaves—from 0.9 to 13.8 mg/g DM, and in nettle leaves—from 1.2 to 17.7 mg/g DM. According to previous studies, the total chlorophyll content in peppermint ranges: from 0.155 to 1.028 mg/g FM (acetone extraction) [57], from 1.39 to 1.69 mg/g FM (80% acetone extraction) [58], from 2.33 to 2.79 mg/g DM (acetone/ammonia blend extraction) [59], from 0.63 to 0.89 mg/g DM (80% ethanol extraction) [45]. According to the literature data, the content of chlorophyll in nettle is in the range from 5.83 to 11.16 mg/g DM (80% methanol extraction) [60] and 4.66–11.26 mg/g DM (96% ethanol extraction) [53]. The results obtained in our study are within the range of data available in the literature.

### 3.2. Antioxidant Properties of the Extracts

SET methods are simple techniques for determining the antioxidant potential. The most popular method is the DPPH method, although some studies have shown that it is better to use FRAP [61]. As demonstrated by Rajurkar et al., the FRAP method also shows a higher linear correlation between the sum of polyphenols and antioxidant properties when compared to the ABTS method [62].

The activity of extracts from peppermint was higher than the activity of extracts from common nettle. In the case of peppermint, the highest activity was characteristic of 30% glycerol–water extracts which, compared with water extracts, had activity levels enhanced by 10–15%. The antioxidant activity of the peppermint and nettle extracts was varied, which can be attributed to the concentrations of bioactive compounds and, in particular, to the concentration of polyphenols.

Glycerol systems allowed for obtaining a higher antioxidant potential of extracts thanks to an increase in the content of water in the extraction mixture (Table 4). For both of the herbs, anhydrous ethanol extracts were characterised by the lowest value of FRAP activity. In other studies, the authors demonstrated that extracts obtained from peppermint with the use of ethanol, methanol and acetone were characterised by higher antioxidant properties at 50% dilution, compared with the same extraction solvents at concentration of 75% [63], with a positive correlation between the polarity of the solvents and the antioxidant properties of the extracts [64]. In the presented study, in the case of the nettle extracts, we observed that the 30% glycerol extract (50 °C) and the 65% glycerol extract (80 °C) were characterised by the highest activity. It should be emphasised that the glycerol–water extracts (from 30% to 65%) have upper range values of antioxidant activity and can be competitive in this respect with extracts obtained with the use of the conventional solvents.

In the presented experiment, the maximum temperature applied for extraction was 80 °C, and that temperature was the optimum for the extraction of polyphenols and flavonoids, while in the case of chlorophyll, the highest extraction efficiency was noted in the case of the temperature of 50 °C, which is consistent with the previously described positive correlation between the temperature of the extraction process and the extraction efficiency [3,4,5,6,7,8]. Extractions conducted at temperature of 20 °C were characterised by significantly lower levels of efficiency in the range of obtained concentrations of active compounds. 

One should also take into account the kind of solvent used and the synergism between the factors, i.e., the extraction system and the temperature. At the temperature of 20 °C, glycerol is characterised by a high density, 1260 kg/m^3^, which affects the process of diffusion, hence inhibiting the extraction of substances from plant material. With increase of temperature, the density of glycerol decreases significantly. This causes a significant increase of extraction efficiency. In addition, the viscosity of glycerol is considerably higher than that of water, which brings about certain technological problems, e.g., during filtration [11].

Another important factor that has an impact on extraction is the polarity of the solvent. With increase of the polarity of the solvent, there is an increase in the efficiency of extraction of phenolic compounds [63]. The results of the presented study indicate that an increase of the content of water in the glycerol–water systems contributed to an increase of concentration of polyphenolic compounds in the extracts from leaves of peppermint and common nettle. In addition, the ethanol–water systems (50%) were characterised by a significantly higher extraction efficiency for polyphenols, which have an affinity for polar solvents, relative to anhydrous ethanol.

In another study, researchers demonstrated that the least efficient solvents for phenolic compounds are those with the lowest polarity, which confirms the increase of obtained concentrations of polyphenols with increase of the polarity of the solvent [64]. With increase of water content in the solutions of acetone, ethanol and methanol, there was an increase of the efficiency of extraction of polyphenols from leaves of black tea and yerba mate [65]. The content of water in ethanol solutions was also the primary factor augmenting the extraction of polyphenols from orange skin [66]. It should also be emphasized that the properties of plant matrices (e.g., uneven distribution of compounds or high enzymatic activity) may make extraction difficult because they inhibit diffusion of compounds, cause simultaneous extraction of other compounds and may even engender transformations of these compounds [67].

Flavonoids and chlorophylls are compounds with a more intense hydrophobic character, and, therefore, such solvents as methanol, ethanol and acetone are relatively better extraction solvents in the anhydrous form than in water solutions [6,63,68,69]. Moreover, interaction between ethanol and flavonoids probably takes place through non-covalent effects, causing diffusion in the solution [70].

In this study, the highest concentrations of flavonoids and chlorophylls were observed in the case of application of anhydrous ethanol systems. In the literature, one can note that higher efficiencies in the concentration of chlorophyll extracted from leaves of common nettle were obtained in the case of ethanol extracts rather than in corresponding water extracts [71]. It was also demonstrated that the efficiency of extraction increased with increasing concentration of ethanol in the solution [71].

When assessing the conditions for the extraction of polyphenols and flavonoids, it can be seen that the isolation efficiency of these groups of compounds depends on the extraction parameters. Azahar et al., optimized the extraction of phenols and flavonoids in the leaves of *Curcuma zedoaria*, although the highest content of flavonoids was found in the extraction at 65–70 °C for 90–100 min, while the highest content of phenols was at extraction at 75–80 °C for a similar extraction time of 80–100 min [72].

What is more, it should be underlined that systems with an addition of glycerol, especially in the case of extracts from peppermint, were generally characterised by higher concentration of flavonoids relative to water extracts (Table 2). This outcome can be attributed to the fact that glycerol has lower polarity than water, so that, in mixtures with water, the dielectric constant of such systems changes, and this ensures more efficient extraction [11].

The constant growth of the market for diet supplements has created a demand for semi-products with high concentrations of biologically active substances obtained, e.g., as a result of the process of extraction, thus posing new challenges for the pharmaceutical and food industries. Summing up the results obtained in the experiment, one can propose glycerol–water systems for the acquisition of alternative extracts from plant materials, which could be used for the production of pharmaceutical preparations, cosmetics or food supplements.

A convenient form of dietary supplements is a liquid form, requiring the use of an appropriate solvent. Water, ethanol and vegetable oils are the most commonly used food grade solvents for the extraction of biologically active substances. Depending on the chemical nature of the solvent, the extraction process results in an extract containing the required fractions of active compounds: hydro- or lipophilic. Unfortunately, water used for extraction of the plant raw material does not have stabilizing properties; therefore, all kinds of water extracts have a short shelf life. On the other hand, ethanol has stabilizing properties, but, due to the limitations of its use for children, people with alcoholism or other diseases that prevent alcohol consumption, it is not always a desirable extractant [14].

### 3.3. Determination of Optimum Conditions of Extraction

Table 5 was compiled to present the lower, medium and upper limits of desirability and the approximated values and percentage difference between the approximated values and the lowest assayed values of the analysed parameters. As can be seen from Table 5, there is a distinct difference between the temperature of extraction and the minimum and maximum values of the determined parameters. As presented in Table 6, temperature had an impact on the optimum composition of the extraction mixture. 

The glycerol/water mixture is a suitable alternative to the typical solvents used in the extraction of biologically active compounds from a plant matrix. Glycerol has a lower dielectric constant compared to water; therefore, its addition to the extraction can increase the efficiency of the process. Karakashov et al. [73] showed in their research that the addition of 10% glycerol (*w*/*v*) increased the content of polyphenols in the extract of *Hypericum triquetrifolium* compared to the aqueous extract of this raw material. The authors obtained a 17.3% increase in TPC content using water with the addition of glycerol [73]. The use of a glycerol/water mixture as a bio-solvent allows for obtaining extracts with high antioxidant activity using an increased extraction temperature. Optimization of polyphenol extraction carried out by Shehata et al. [13] indicated that glycerol concentration up to 90% (*w*/*v*) ensures high content of this group of compounds in extracts from two species of Artemisia [13]. The optimization of polyphenol extraction from olive leaves described by Mourtzinos et al. [74] demonstrated that lowered glycerol concentration and lower extraction temperature can be achieved by using the addition of cyclodextrin as a performance enhancing substance. The authors obtained optimal extraction parameters using 60% (*w*/*v*) glycerol with 7% (*w*/*v*) cyclodextrin addition and conducting the experiment at a temperature of 60 °C [74]. Differences between the optimal extraction conditions for peppermint leaves and nettle leaves may result from their different phytochemical composition. Benchikh and Louailèche [75] showed a linear relationship between temperature and TPC and TFC content for carob (*Ceratonia siliqua* L.) pulp extracts, while Alide et al. [76] showed that the extraction efficiency of polyphenolic compounds from garlic (*Allium sativum* L.) may depend on the extraction temperature and the polarity of the extractant. When water was used as an extractant, the content of TPC and TFC increased with increasing temperature, while extraction with ethanol under the same conditions caused a decrease in the extraction efficiency of these groups of compounds [76]. The use of statistical modelling allowed researchers to determine the utility profile in order to obtain the optimal content of polyphenols (TPC and TF), chlorophyll and the highest antioxidant activity for the extracts of peppermint leaves and nettle leaves using the optimal composition of glycerol/water extractant.

To obtain the highest values of the analysed parameters, leaves of peppermint should be extracted with glycerol–water mixture at the proportions of 30.5:69.5 at temperature of 50 °C, while the optimal conditions for the extraction of leaves of common nettle were the glycerol–water proportions of 12.5:87.5 and extraction temperature of 20 °C. Obtaining different optimal extraction parameters for individual tested plant materials indicates the significant influence of the type of plant matrix [64]. In the optimal conditions of extraction, the approximated values (PV) for both raw materials were only slightly lower than the maximum values obtained experimentally. Comparing the average percentage differences between the highest values of the analysed parameters obtained in the experiment and the approximated values for various temperatures (Table 5, PV%) with the level of desirability (Table 6, desirability level), one can note a high correlation, which, in the analysed examples, amounted to 0.9681.

The applied method of optimization of the extraction parameters with the use of statistical modelling can be used in industry in pre-formulation studies in order to obtain the most useful parameters.

## 4. Conclusions

Raw materials of plant origin are a rich source of antioxidant phytochemicals that can be used in various ways in food and medicine. Currently, safe and natural solvents are being sought for the extraction of active compounds from plant matrices, in line with the principle of "green chemistry". Our experimental results demonstrate that glycerol can be used as an extractant. The presented study showed that 30.5% and 12.5% glycerol solutions were the optimal solvents for the extraction of polyphenolic compounds, flavonoids and chlorophyll from peppermint leaves and nettle leaves, respectively. Temperature was also found to influence the antioxidant activity and the extraction efficiency of active compounds from the tested raw materials. In the case of mint leaves extraction, the optimal utility profile values were obtained at temperatures of 50 °C > 80 °C > 20 °C, and for nettle leaves, this was 20 °C > 80 °C > 50 °C. The significant influence of the type of plant matrix was made evident in the various optimal extraction parameters (extraction temperature and the composition of the solvent system) for individual tested plant materials. The novelty of the presented work is the optimization of the conditions for the extraction of polyphenols, flavonoids and chlorophyll from the studied plant materials with the use of glycerol, which was carried out for the first time.

## Figures and Tables

**Table 1:** Solvent content of the extraction systems used in the experiment.

Extraction System	The Solvent Content in the Extraction System [%]	
	Glycerol (x_1_)	Water (x_2_)
W	0	100
G 30	30	70
G 50	50	50
G 65	65	35
G 80	80	20

W—water, G—glycerol.

**Table 2:** Content of polyphenols, flavonoids and chlorophyll in extracts from peppermint.

Temperature [°C]	Extraction System ^1^	Polyphenols		Flavonoids		Chlorophyll	
		[mg GAE/L] ^2^ (Y_1_)	[mg GAE/g DM] ^3^	[mg QE/L] (Y_2_)	[mg QE/g DM]	[mg/L] (Y_3_)	[mg/g DM]
**Water and Glycerol–Water System**							
80	W	387.7 ± 25.6 ^abc^	19.4	62.3 ± 3.6 ^ef^	3.1	54.0 ± 2.5 ^i^	2.7
	G 30	412.5 ± 32.7 ^a^	20.6	107.2 ± 5.6 ^c^	5.4	84.4 ± 4.1 ^e^	4.2
	G 50	372.4 ± 22.0 ^bcd^	18.6	114.2 ± 2.4 ^c^	5.7	73.2 ± 3.4 ^f^	3.7
	G 65	340.7 ± 22.9 ^cde^	17.0	114.5 ± 6.8 ^c^	5.7	70.2 ± 2.9 ^fg^	3.5
	G 80	276.7 ± 21.8 ^hi^	13.8	113.4 ± 5.0 ^c^	5.7	64.3 ± 2.6 ^h^	3.2
50	W	357.1 ± 23.8 ^bcd^	17.9	51.7 ± 2.8 ^f^	2.6	88.0 ± 4.1 ^e^	4.4
	G 30	389.1 ± 25.0 ^ab^	19.5	86.1 ± 4.4 ^d^	4.3	87.2 ±3.9 ^e^	4.4
	G 50	355.0 ± 28.1 ^bcd^	17.8	80.8 ± 4.6 ^d^	4.0	89.0 ±4.7 ^e^	4.5
	G 65	316.0 ± 24.7 ^efghi^	15.8	65.9 ± 3.7 ^e^	3.3	50.3 ±1.8 ^i^	2.5
	G 80	197.6 ± 16.8 ^j^	9.9	55.8 ± 3.3 ^ef^	2.8	51.6 ±2.5 ^i^	2.6
20	W	310.9 ± 21.4 ^fghi^	15.5	49.5 ± 3.2 ^f^	2.5	50.1 ± 1.4 ^i^	2.5
	G 30	316.9 ± 23.4 ^efg^	15.8	81.1 ± 4.5 ^d^	4.1	66.6 ± 3.1 ^gh^	3.3
	G 50	274.6 ± 29.8 ^i^	13.7	50.3 ± 2.5 ^f^	2.5	23.8 ± 0.9 ^j^	1.2
	G 65	155.2 ± 11.2 ^k^	7.8	36.2 ± 2.1 ^g^	1.8	24.8 ± 1.3 ^j^	1.2
	G 80	152.1 ± 9.5 ^k^	7.6	32.3 ± 2.3 ^g^	1.6	18.9 ± 0.7 ^k^	0.9
**Ethanol and Ethanol–Water System**							
50	E	40.7 ± 2.9 ^l^	2.0	199.8 ± 12.7 ^a^	10.0	276.2 ± 14.3 ^a^	13.8
	E 50	319.6 ± 25.5 ^efg^	16.0	118.5 ± 6.7 ^c^	5.9	127.1 ± 6.6 ^c^	6.4
20	E	29.5 ± 1.7 ^m^	1.5	150.3 ± 9.3 ^b^	7.5	201.3 ± 9.4 ^b^	10.1
	E 50	286.2 ± 21.6 ^ghi^	14.3	83.2 ± 4.5 ^d^	4.2	101.5 ± 4.3 ^d^	5.1

W—water, G—glycerol, GAE—gallic acid equivalent, QE—quercetin equivalent. ^1^ Sample code—designations as in Table 1; ^2^ component concentration in the liquid extract; ^3^ the content of ingredients in the dry matter of the plant raw material; ^a–l^—identical lowercase letters indicate significant difference (*p* > 0.05).

**Table 3:** Content of polyphenols, flavonoids and chlorophyll in extracts from common nettle.

Temperature [°C]	Extraction System ^1^	Polyphenols		Flavonoids		Chlorophyll	
		[mg GAE/L] ^2^ (Y_1_)	[mg GAE/g DM] ^3^	[mg QE/L] (Y_2_)	[mg QE/g DM]	[mg/L] (Y_3_)	[mg/g DM]
**Water and Glycerol–Water System**							
80	W	113.9 ± 9.0 ^d^	5.7	53.6 ± 2.8 ^e^	2.7	130.3 ± 6.0 ^f^	6.5
	G 30	132.3 ± 10.5 ^bc^	6.6	63.4 ± 3.3 ^d^	3.2	151.4 ± 7.3 ^e^	7.6
	G 50	140.3 ± 11.8 ^bc^	7.0	50.6 ± 2.4 ^e^	2.5	115.1 ± 5.0 ^g^	5.8
	G 65	150.9 ± 12.1 ^ab^	7.5	50.0 ± 2.9 ^ef^	2.5	83.6 ± 3.9 ^h^	4.2
	G 80	74.7 ± 5.5 ^gh^	3.7	42.5 ± 2.1 ^hi^	2.1	50.7 ± 2.1 ^j^	2.5
50	W	120.3 ± 8.6 ^bcd^	6.0	51.1 ± 2.9 ^ef^	2.6	170.2 ± 8.1 ^d^	8.5
	G 30	161.8 ± 11.7 ^a^	8.1	61.2 ± 3.4 ^d^	3.1	190.9 ± 9.2 ^c^	9.5
	G 50	136.9 ± 10.1 ^bc^	6.8	49.2 ± 2.7 ^ef^	2.5	83.2 ± 3.0 ^h^	4.2
	G 65	84.5 ± 5.6 ^fg^	4.2	47.0 ± 2.2f ^g^	2.4	58.2 ± 2.1 ^i^	2.9
	G 80	65.3 ± 4.5 ^hi^	3.3	42.0 ± 2.3 ^hi^	2.1	57.5 ± 2.9 ^i^	2.9
20	W	91.4 ± 6.5 ^f^	4.6	47.6 ± 2.2 ^fg^	2.4	130.1 ± 6.6 ^f^	6.5
	G 30	100.3 ± 7.4 ^ef^	5.0	45.2 ± 1.9 ^gh^	2.3	148.7 ± 7.1 ^e^	7.4
	G 50	78.3 ± 5.8 ^g^	3.9	37.8 ± 2.7 ^i^	1.9	55.8 ± 2.2 ^i^	2.8
	G 65	63.6 ± 4.9 ^i^	3.2	29.6 ± 1.6 ^j^	1.5	36.5 ± 2.0 ^k^	1.8
	G 80	48.3 ± 3.4 ^j^	2.4	13.6 ± 0.9 ^k^	0.7	24.5 ± 1.1 ^l^	1.2
**Ethanol and Ethanol–Water System**							
50	E	31.9 ± 2.1 ^k^	1.6	102.7 ± 5.4 ^a^	5.1	354.7 ± 19.9 ^a^	17.7
	E 50	109.0 ± 7.5 ^de^	5.5	89.8 ± 4.0 ^b^	4.5	146.8 ± 8.1 ^e^	7.3
20	E	22.3 ± 1.6 ^l^	1.1	75.6 ± 4.1 ^c^	3.8	297.7 ± 13.4 ^b^	14.9
	E 50	77.9 ± 5.6 ^g^	3.9	37.6 ± 2.1 ^i^	1.9	120.0 ± 6.3 ^fg^	6.0

W—water, G—glycerol, GAE—gallic acid equivalent, QE—quercetin equivalent. ^1^ Sample code—designations as in Table 1; ^2^ component concentration in the liquid extract; ^3^ the content of ingredients in the dry matter of the plant raw material; ^a–l^—identical lowercase letters indicate significant difference (*p* > 0.05).

**Table 4:** FRAP antioxidant activity for extracts from peppermint and common nettle.

Temperature [°C]	Extraction System ^1^	FRAP [μmol Fe^2+^/L] (Y_4_)	
		Leaf of Peppermint	Leaf of Common Nettle
**Water and Glycerol–Water System**			
80	W	8494 ± 159 ^b^	4030 ± 82 ^e^
	G 30	10075 ± 228 ^a^	4587 ± 92 ^cd^
	G 50	7270 ± 144 ^c^	4718 ± 93 ^c^
	G 65	6635 ± 129 ^d^	5945 ± 119 ^b^
	G 80	4222 ± 79 ^g^	3211 ± 61 ^g^
50	W	7160 ± 148 ^c^	4460 ± 65 ^d^
	G 30	8625 ± 191 ^b^	6736 ± 63 ^a^
	G 50	7208 ± 132 ^c^	4016 ± 86 ^e^
	G 65	5760 ± 153 ^e^	3021 ± 76 ^hi^
	G 80	4349 ± 161 ^g^	2388 ± 69 ^k^
20	W	5165 ± 128 ^f^	3180 ± 104 ^gh^
	G 30	5761 ± 162 ^e^	3540 ± 91 ^f^
	G 50	4373 ± 168 ^g^	2912 ± 88 ^ij^
	G 65	3366 ± 55 ^h^	2827 ± 65 ^j^
	G 80	2909 ± 49 ^i^	2493 ± 58 ^k^
**Ethanol and Ethanol–Water System**			
50	E	2283 ± 81 ^j^	1633 ± 38 ^l^
	E 50	5913 ± 143 ^e^	4164 ± 91 ^e^
20	E	1600 ± 41 ^k^	1178 ± 29 ^m^
	E 50	4580 ± 149 ^g^	2838 ± 71 ^j^

W—water, G—glycerol; ^1^ Sample code—designations as in Table 1. ^a–l^—values designated with the same letters within columns do not significantly differ at 5% error.

**Table 5:** Optimum conditions of extraction.

	Leaf of Peppermint					Leaf of Common Nettle				
Desirability	L_i_ (0)	M_i_ (0.5)	U_i_ (1)	PV	PV [%]	L_i_ (0)	M_i_ (0.5)	U_i_ (1)	PV	PV [%]
TPC 80	276.7	344.6	412.5	401.0	97.21	74.5	112.7	150.9	148.2	98.21
TF 80	62.3	88.4	114.5	109.7	95.81	42.5	53.0	63.4	58.6	92.43
CHLO 80	54.0	69.2	84.4	78.9	93.48	50.7	101.1	151.4	141.2	93.26
FRAP 80	4222	7149	10075	9020	89.53	3211	4578	5945	5097	85.74
TPC 50	197.6	293.6	389.1	396.1	101.80	65.3	113.6	161.8	150.5	93.02
TF 50	51.7	6.8.9	86.1	81.9	95.12	42.0	51.6	61.2	56.3	91.99
CHLO 50	50.3	69.7	89.0	88.6	99.55	55.7	123.3	190.9	160.6	84.13
FRAP 50	4349	6487	8625	8202	95.10	2388	4562	6736	5563	82.59
TPC 20	152.1	234.5	316.9	316.9	100.00	48.3	74.3	100.3	96.3	96.01
TF 20	32.3	56.7	81.1	64.8	79.90	13.6	30.6	47.6	46.4	97.48
CHLO 20	18.9	42.8	66.6	52.8	79.28	24.5	86.6	148.7	132.3	88.97
FRAP 20	2493	3017	3540	3344	94.46	2493	3017	3540	3328	94.01

Abbreviation: Limits: L_i_—low; M_i_—mean; U*_i_*—upper; PV—predicted values; PV%—percentage of U_i_.

**Table 6:** Optimum conditions of extraction with a glycerol:water mixture and the level of desirability.

Tested Raw Material	Extraction Temperature [°C]	Glycerol [%]	Water [%]	Desirability Level
Leaf of Peppermint	80	37.4	62.6	0.86
	50	30.5	69.5	0.94
	20	18.6	81.4	0.79
Leaf of Common Nettle	80	31.8	68.3	0.82
	50	21.6	78.4	0.78
	20	12.5	87.5	0.89

## Data Availability

The data used to support the findings of this study are included.
